# Role of Vitamin D in Celiac Disease and Inflammatory Bowel Diseases

**DOI:** 10.3390/nu14235154

**Published:** 2022-12-03

**Authors:** Claudia Infantino, Roberta Francavilla, Adriana Vella, Sabrina Cenni, Nicola Principi, Caterina Strisciuglio, Susanna Esposito

**Affiliations:** 1 Department of Medicine and Surgery, Pediatric Clinic, University of Parma, 43126 Parma, Italy; 2 Department of Woman, Child and General and Specialized Surgery, Second University of Naples, 80138 Naples, Italy; 3 Università degli Studi di Milano, 20122 Milan, Italy

**Keywords:** celiac disease, inflammation, inflammatory bowel diseases, microbiota, vitamin D

## Abstract

Vitamin D (VD) is a pro-hormone that has long been known as a key regulator of calcium homeostasis and bone health in both children and adults. In recent years, studies have shown that VD may exert many extra-skeletal functions, mainly through a relevant modulation of the innate and adaptive immune system. This has suggested that VD could play a fundamental role in conditioning development, clinical course, and treatment of several autoimmune disorders, including celiac disease (CD) and inflammatory bowel diseases (IBDs). The main aim of this review is to evaluate the relationships between VD, CD, and IBDs. Literature analysis showed a potential impact of VD on CD and IBDs can be reasonably assumed based on the well-documented in vitro and in vivo VD activities on the gastrointestinal tract and the immune system. The evidence that VD can preserve intestinal mucosa from chemical and immunological damage and that VD modulation of the immune system functions can contrast the mechanisms that lead to the intestinal modifications characteristic of gastrointestinal autoimmune diseases has suggested that VD could play a role in controlling both the development and the course of CD and IBDs. Administration of VD in already diagnosed CD and IBD cases has not always significantly modified disease course. However, despite these relevant problems, most of the experts recommend monitoring of VD levels in patients with CD and IBDs and administration of supplements in patients with hypovitaminosis.

## 1. Introduction

Vitamin D (VD) is a pro-hormone that has long been known as a key regulator of calcium homeostasis and bone health in both children and adults [1]. In recent years, studies have shown that VD may exerts many extra-skeletal functions, mainly through a relevant modulation of the innate and adaptive immune system [2]. This has suggested that VD could play a fundamental role in conditioning development, clinical course, and treatment of several autoimmune disorders, including celiac disease (CD) and inflammatory bowel diseases (IBDs) [3,4,5]. The evidence that VD could directly protect the intestinal wall, modulating the expression of tight junctions (TJ) and controlling antimicrobial peptide synthesis has further highlighted the importance of VD in the onset and control of gastrointestinal immune disorders [6]. However, mainly due to this very fast-growing scientific area, several aspects of the relationships between VD, CD, and IBDs remain surrounded by uncertainties, without a final agreement having been reached. This, at least in part, depends on the incomplete knowledge of some aspects of VD availability and requirement, definition and evaluation of VD deficiency, and direct and indirect effect of VD on the whole body [7]. Consequently, monitoring of serum VD values in CD and IBSs remains debated as is the need of VD in patients with these diseases. Discussion on the present knowledge on these topics is the main aim of this review.

An in-depth research and review of the medical literature was performed. The MEDLINE–PubMed database was searched from 2014 to 2022 to collect the literature. The search included randomized placebo-controlled trials, controlled clinical trials, double-blind, randomized controlled studies, and systematic reviews of the last five years. The following combinations of keywords were used: “vitamin D” AND “inflammation” AND/OR “inflammatory bowel diseases” AND/OR “IBD” AND/OR “celiac disease” AND “children” OR “paediatric” OR “pediatric” OR “adolescent”. We also performed a manual search of the reference lists of the obtained studies. The search was limited to English-language journals and full papers only.

## 2. Sources, Synthesis, and Serum Vitamin D Levels

Two major forms of VD exist: VD2 (ergocalciferol) and VD3 (cholecalciferol). VD2 is contained in some foods, mainly the flesh of fatty fish and yeast, sun dried and ultraviolet irradiated mushrooms, and plants [8]. The dietary intake of VD2 is generally too low to assure the minimum VD requirement coverage. As a consequence, several commercial foods, especially those for infants and young children, are fortified with VD. However, independently from diet, people get most of the VD from VD3 that is produced in the skin from 7-dehydrocholesterol, an intermediate in cholesterol synthesis, after exposure to ultraviolet B (UVB) light of the sun [9]. The dose response for dermal photosynthesis of VD increases linearly at small UV doses but reaches a plateau well below the threshold dose for erythema. This means that short but frequent exposures to the sun induce a greater dermal synthesis of VD than after single exposures of the same overall duration. However, together with type and duration of sun exposure, several other factors, such as age, skin color, sunscreen use, latitude, time of day, and season may influence skin VD synthesis [9,10]. As a result, recommendations regarding exposure to sunlight in order to maintain adequate VD concentration differ significantly between scientific societies [10,11].

Both VD forms enter the circulation mainly bound to the VD-binding protein (VDBP). Only about 15% of them are bound to albumin, and only about 0.03% are free. Bound VD2 and VD3 are transported to the liver, where they are transformed in the 25-hydroxy-vitamin D [25(OH)D] by CYP2R1 and other enzymes and later to the kidney, where a second hydroxylation through the VD-activating enzyme 1-α-hydroxylase (CYP27B1) takes place [12]. This leads to the synthesis of the active VD, the 1,25- dihydroxy-vitamin D [1,25(OH)2D], also known as calcitriol. In addition, several extra-renal cells harbor CYP27B1 and contribute to actively metabolize VD and convert 25(OH)D to 1,25(OH)2D at the local tissue level, playing a fundamental role in the global activity of VD [13]. In the cellular targets, the activated VD binds to the nuclear VD receptor (VDR) and forms a heterodimeric complex with the retinoic acid X receptor that recognizes specific DNA sequences known as VD responsive elements (VDRE), leading to the regulation of the transcription of over 1000 target genes [14]. VDR is widely expressed in various tissues (i.e., skin, parathyroid gland, adipocyte, small intestines, and colon) and throughout the immune system [15], and its expression regulates final VD activity [16].

Despite calcitriol being the active VD form, serum concentration of 25(OH)D is currently the main indicator of VD status. This is because 1,25(OH)2D circulates in very small amounts; serum levels are tightly regulated by the parathyroid hormone, calcium, and phosphate, and the half-life is very short as it is measured in hours [17]. Moreover, levels of 1,25(OH)2D do not decrease until VD deficiency is severe. In contrast, 25(OH)D can be detected in significantly greater amounts, has a circulating half-life of 15 days, and its concentrations are less dependent on mineral content and parathyroid hormone activity [18]. However, assessing VD status by measuring total serum 25(OH)D concentrations is complicated by the considerable variability of the available assays (i.e., immunoassays, HPLC, and mass spectrometry) used to evaluate this compound [17,18]. Further limitations can derive from the evidence that measuring total 25(OH)D levels may be misleading in conditions that alter VDBP concentrations, such as liver diseases, or modify its binding to 25(OH)D, such as in patients with mutations in the VDBP gene [19]. Finally, as methods also measure inactive VD metabolites, such as 24,25-dihydroxyvitamin D and the 3-epimer of 25(OH)D, dosages may overestimate total 25(OH)D concentrations [20]. All these factors preclude the ability to pool research data from different studies and to allow evidence-based definitions of VD status. This explains why, despite several attempts to standardize laboratory assays and correlate clinical manifestation with 25(OH)D serum concentrations having been made [21,22], it is still controversial what level of 25(OH)D is optimal for overall health benefits, how much VD is needed to maintain health and whether there are differences for the various potentially preventable diseases, how VD should be administered (i.e., daily or with bolus doses), and which are the toxic doses. Different definitions of deficiency, inadequacy, and toxicity of VD intake, together with different recommended dietary allowances, have been suggested by the various scientific societies. The Food and Nutrition Board (FNB) at the National Academies of Sciences, Engineering, and Medicine (NASEM) of the USA has stated that for adults VD deficiency and inadequacy occur when serum 25(OH)D concentrations are <2 ng/mL and between 12 and 20 ng/mL, respectively [23]. Concentrations ≥20 ng/mL were considered adequate for maintaining health although those >50 ng/mL could be associated with the development of adverse events. In contrast, the Endocrine Society of the USA stated that for clinical practice a serum 25(OH)D concentration >30 ng/mL was necessary to maximize the effect of VD bone and muscle metabolism [24]. Despite adequate studies establishing an intake–response association between VD and the physiological outcome not being available, NASEM has suggested that the Recommended Dietary Allowance (RDA) for VD could be 600 IU per day for people older than 1 year and 400 IU for children <1 year. The same recommendations were published by the European Food Safety Authority (EFSA), considering that this intake could be high enough to reach serum 25(OH)D concentrations of at least 20 ng/mL [25]. Particularly for children, for whom an adequate VD intake is essential for bone growth and development, these recommendations have been reanalyzed several times. Evidence was considered insufficient for setting established definitive recommendations for children in the first half-year of life, but it was noted that 400 IU per day were considered adequate for the majority of infants at this age [26,27]. Moreover, a review of the study that has measured the effect of daily VD supplementation on 25(OH)D serum levels revealed that each 100/IU per day increase in VD supplementation was associated with an average of 0.8 ng/mL increase in 25(OH)D concentration [28]. Moreover, it was calculated that the tolerable upper VD intake levels were 1000 IU, 1500 IU, and 2500 IU per day for children 0–6 months, 7–12 months, and 1–4 years of age, respectively [28]. For adults, values vary according to subject characteristics: in healthy people, tolerable upper intake VD level is 4000 IU per day; in people at-risk, it increases to 10000 IU per day [23,24].

## 3. Vitamin D Role in Preserving the Integrity of the Intestinal Epithelial Barrier

Compromise or disruption of the intestinal barrier function causes increased intestinal permeability that results in exposure of the host to luminal antigens and bacteria, leading to a break of tolerance and onset of inflammation [29]. It is suggested that VD can assure intestinal mucosa integrity through mechanisms such as regulation of colonic mucus [30] and preservation of intestinal wall structure [31]. However, most of the studies in this regard seem to indicate that the role of VD is mainly played by its influence on gut microbiota composition and functions [32] and its ability to upregulate the TJ protein expression and to significantly suppress the release of zonulin, a family of molecules that are responsible for the structural disassembly of TJ and the increase in intestinal permeability [33]. Table 1 summarizes the VD mechanisms in preserving the integrity of the intestinal epithelial barrier.

Gut microbiota composition is critical to maintain normal intestinal functions. In most gastrointestinal diseases, including CD and IBDs, gut dysbiosis is common, with a significant increase in the concentrations of pathogenic bacteria (*Enterobacteriaceae* and *Fusobacterium*) and reduction in the presence of those with documented beneficial activity (*Bifidobacteria, Faecalibacterium prausnitzii*, and *Lactobacilli* strains) [34,35]. This is associated with a potential block of the immune responses potentiated by VD, including VDR expressions and related immune functions of activated VD [32]

TJs are made by several proteins, including claudins, occludin, junctional adhesion molecules, and zonula occludens (ZO) proteins. Their main functions are to prevent leakage of solutes and water sealing the space between adjacent epithelial cells, preventing increased permeability of the intestinal mucosa. Several in vitro and in vivo studies have shown that VD protects TJs from the damage due to chemical exposure such as those due to dextran sulfate sodium and alcohol exposure [36,37]. Moreover, it reduced the TNF-α induced injury [38]. Finally, a number of studies have shown that VD/VDR upregulates TJ expression in human epithelium cells and that in experimental animals VDR deletion in intestinal epithelial cells is associated with a significant decrease of some TJ expression [39,40,41].

## 4. Immunomodulating Activity of Vitamin D

**Figure 1:**
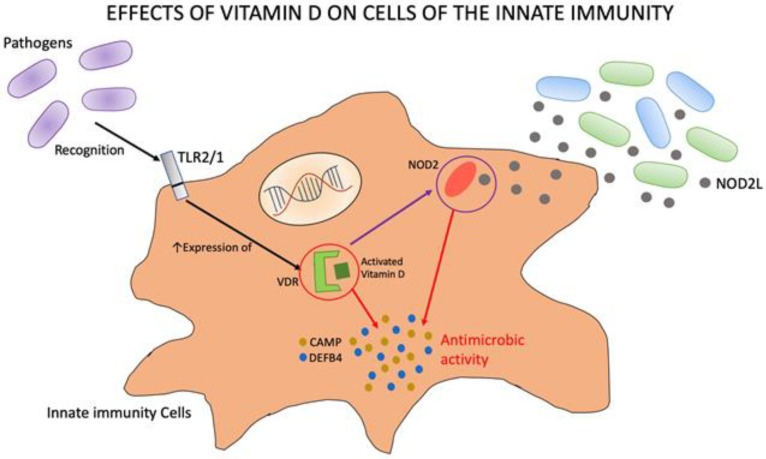
How vitamin D interacts with cells of the innate immunity.

Cells of the immune systems, such as macrophages, dendritic cells, monocytes, and T and B cells, harbor CYP27B1 and express VDR. Direct production of 1,25(OH)2D by these cells results in a significant modulation of both innate and adaptive immunity. Regarding innate immunity, it has been reported that activated VD stimulates the production by neutrophils, macrophages, and cells lining epithelial surfaces of antibacterial peptides with broad antimicrobial activity, such as cathelicidin (CAMP) and β-defensin 2 (DEFB4) [42,43,44]. Interestingly, this kind of VD-associated increase in innate immunity is strictly dependent on toll-like receptor 2/1 (TLR2/1) heterodimers activity and become maximal in case of need. When TLR2/1 recognizes cell membranes of certain microbes, it stimulates expression of VDR, significantly increasing the antimicrobial effect against pathogens [45]. Moreover, VD induces the intracellular pathogen recognition receptor NOD2 that increases cell sensitivity to NOD2 ligan produced by certain bacteria. This enhances transcription of CAMP and DEFB4 with a further increase in antimicrobial activity. Finally, VD suppresses hepcidin anti-microbial peptide expression, reducing ferroportin-mediated export of intracellular iron and limiting proliferation of pathogens that utilize iron for growth [46]. Figure 1 shows the effects of VD on cells of the innate immunity.

Regarding antigen presenting cells and adaptive immunity, VD acts on dendritic cells (DC), regulating their differentiation and maturation and favoring the development of a tolerogenic phenotype. Exposure of monocytes to 1,25(OH)2D increases the expression of molecules involved in antigen capture and inhibits DC differentiation and maturation with reduced stimulatory capacity for the antigen-specific CD8 T cells. Peripheral pro-inflammatory Th1 response is reduced. Moreover, VD increases T regulatory cells, limits the number of CD4+ T cells, upregulates IL-10, and reduces tumor necrosis factor-α (TNF-α) and interferon γ (IFN-γ) levels. Practically, VD shifts the immune response from a pro-inflammatory Th1 response to an anti-inflammatory Th2 response, increasing the secretion of IL-4 while decreasing the secretion of interleukin (IL)-2 and IFN-γ [47].

Table 2 summarizes immunomodulating activity of VD on immune systems.

## 5. Vitamin D and Celiac Disease

### 5.1. Epidemiology and Developmental Factors

CD is a chronic, systemic autoimmune disease triggered by the exposure to dietary gluten proteins in genetically predisposed individuals, mainly those positive for human leucocyte antigen HLA DQ2 or DQ8 [48]. The evidence that in genetically predisposed subjects the chronic exposure to dietary gluten is invariably accompanied by the production of autoantibodies against transglutaminase 2 (TG2), an enzyme that plays a critical role for gluten induced pathogenesis in CD, is the best evidence of the autoimmune origin of this disease [49].

For years, CD has been considered only a gastrointestinal disease. It was thought that the severe intestinal mucosa alterations (i.e., increase in intraepithelial lymphocytes, crypt hyperplasia, various degrees of villi atrophy, and polymorphic inflammatory infiltrate of the lamina propria) due to the autoimmune process were only associated with chronic diarrhea, malabsorption, and failure to thrive [50,51]. Starting from the 1980s, it was evidenced that in several patients in whom CD could be diagnosed on the base of serology, genetic testing, and duodenal biopsy findings, gastrointestinal manifestations could be lacking or have poor relevance. On the contrary, a number of extraintestinal signs of disease, such as short stature, delayed puberty, recurrent iron deficiency anemia, dental enamel hypoplasia, osteoporosis, liver damage, and neurological problems alone or in association, represented the main elements of the clinical picture of the disease [52]. Presently, about 50% of diagnosed CD do not have classical manifestations and present with a predominance of extra-intestinal symptoms [53]. Moreover, in some patients, signs and symptoms of other autoimmune diseases, including type 1 diabetes, autoimmune thyroiditis, and autoimmune hepatitis, likely due in part to shared genetic factors, are described [54].

Whereas classic CD remains strictly dependent on the direct effect of the autoimmune damage of the intestinal mucosa, the reasons why signs of non-typical cases develop are not precisely defined. It has been supposed that in some patients, such as for example, those with anemia, stunted growth, and osteopenia, manifestations are likely caused by nutrient malabsorption secondary to intestinal mucosa damage [55]. Other conditions, despite evidence being lacking, are supposed to be due to autoimmunity. On the other hand, TG2 is present in almost all cell types and participates in various biological reactions. Consequently, autoantibodies against TG2, systematically detected in all CD patients, have the potential to negatively affect the activity of the enzyme and its biological effects in tissues outside of the gastrointestinal tract [56].

CD is a relatively common disease, and several epidemiological studies indicate that its incidence is increasing worldwide. Previous evaluations have shown a global incidence of about 1.4%, although with slight differences between continents [57]. On the contrary, recent reports have shown that incidence can be even higher than double previously calculated [58,59], with rates increasing by 8.1% per year from 1950 through 2010 [60]. These findings were initially considered an overestimation due to the greater attention paid to CD by physicians and the large use of serology, a method with relatively poor sensitivity, as the only diagnostic test for CD identification [61]. However, the evidence that the increase could be demonstrated even when the combination of serologic test results and duodenal biopsy findings were used for CD diagnosis confirmed that CD rates were really increasing and suggested that other environmental factors other than gluten exposure could favor CD development. Results of recent studies clearly indicate the possible roles of microbiota, time of gluten introduction, delivery method, history of breastfeeding, acute viral gastrointestinal infections, and micronutrient deficiency in the development of CD [62,63].

### 5.2. Relationships between Vitamin D and Celiac Disease Development

Among micronutrients, a role has been ascribed to VD due to its importance in the regulation of both innate and adaptive immune system activity [64]. The evidence that in some conditions, potentially associated VD hypovitaminosis, the risk of CD development is greater has strongly supported this hypothesis. Several studies have found that among people living in northern geographic areas where exposure to sunlight or UVB radiation is lower as is the skin synthesis of VD, CD is more common [65]. Moreover, children born during summer months who receive gluten for the first-time during the winter and are consequently at greater risk of VD deficiency when autoimmunity may develop suffer more frequently from CD than those born during cold months [66]. Finally, in CD patients VD deficiency caused by malabsorption can be the most important cause of some clinical manifestations of the disease, such as reduced bone mineral density, reduced bone mass, and increased bone fragility [67].

VD may interfere with CD development and course through several mechanisms that limit or prevent the intestinal damage due to gluten protein exposure. VD protects the intestinal wall and contrasts the immune response [64]. In CD patients, barrier function as well as TJ are significantly altered [68] as gliadin peptides have a dissociative effect on TJ proteins, namely actin and ZO-1, which leads to increased gut permeability and entrance of gliadin peptides to the lamina propria [69]. VD can also correct the immune alterations that are characteristic of CD. In CD patients, adaptive immunity is altered as after passage of the permeable intestinal mucosa gluten peptides are deamidated by tissue transglutaminase and bind to human leucocyte antigens (HLA-DQ2 and HLA-DQ8) on antigen-presenting cells. CD4+ T-cells are activated, and an inflammatory response occurs due to the secretions of Th1 cytokines that lead to the classic mucosal lesions [70,71,72,73]. As previously reported, VD can significantly influence these immune alterations, reducing T cell stimulatory capacity and reverting Th1 response to a more tolerogenic response [70,71,72,73].

Starting from these considerations, it was presumed that in CD patients with newly onset disease, VD deficiency could be detected, and this could confirm the role of VD in CD pathogenesis. At the same time, the evidence that VD deficiency was common in patients with established CD and VD administration could lead to a more favorable disease course could confirm a role of VD in conditioning CD evolution [67,68,69,70].

### 5.3. Impact of Vitamin D Supplementation on Celiac Disease Course

Unexpectedly, results of clinical studies did not always confirm these hypotheses. Regarding serum VD levels in CD patients, results were controversial. A study carried out by Villanueva et al. did not report any difference in VD concentrations between children with CD and children without [74]. Similar results were reported by Lerner et al., who compared the VD status of CD children with that of children with no specific abdominal pain, their parents, and CD adults [75]. Interestingly, whereas children with and without CD had normal serum VD levels, low serum VD concentrations were detected in adult subjects. The strict age dependency of VD deficiency in CD patients was further suggested by a recent critical review of 27 papers and 28 sets of data concerning 1137 CD patients and 2613 controls [76]. It was shown that considering together all the CD patients regardless of age, these had a significant lower serum 25(OH)D concentrations than controls (21.7 ng/mL vs. 25.24 ng/mL) [76]. However, when age subgroups were considered, it was evidenced that the difference between CD and healthy subjects could be totally ascribed to values found in adult patients as analysis of pediatric data revealed that concentrations detected in these patients were quite like those evidenced in healthy controls (weighted mean difference -0.51, 95% confidence interval [CI] –2.46 to 1.72 nmol/L). Different VD levels in children compared to adults were explained by the common use in this group of subjects of VD fortified foods, the common VD administration in the first years of life to prevent rickets, and the greater exposure to sun. Different results were reported by other studies. Lionetti et al. enrolled 131 children with newly diagnosed CD and a similar number of healthy subjects and found that plasma 25(OH)D levels were significantly lower in patients than in controls (25.3 ± 8.0 and 31.6 ± 13.7 ng/mL; *p* < 0.0001) [77]. This difference was maintained when subgroups of only deficient cases (VD <20 ng/mL) were evaluated (31% among patients vs. 12% in controls; *p* < 0.0001) and when analysis was restricted to blood samples collected during summer (*p* < 0.01) and autumn (*p* < 0.0001). Similar findings were collected in another study in which comparison of 25(OH)D concentrations between 60 children aged 0–18 years diagnosed with CD and 60 healthy age- and sex-matched controls revealed that the prevalence of levels <20 ng/mL was significantly greater in the cases (63.3%) than in the controls (45.0%) [78]. Differences in criteria used to define deficiency, methods of VD dosage, small sample size of enrolled subjects, lack of a control group, retrospective methods of recording, and possible poor consideration of the possible cofactors influencing VD concentrations, such as month of blood testing, ethnicity, or type of diet received by patients, may explain the different results.

No definitive conclusion can also be drawn regarding VD supplementation in CD patients. The current available treatment for CD is a lifelong gluten-free diet (GFD). This leads to significant clinical improvement within a few weeks and to mucosal recovery and healing after a longer time, up to 2 years [79,80]. Progressive normalization of mucosal structure and function during GFD is per se associated with a spontaneous increase in VD serum concentration in CD patients. However, the diet-related increase may not be sufficient to normalize VD serum levels. Verma et al., studying a group of children with newly onset CD, reported that in those with VD insufficiency (serum concentration 12–20 ng/mL) 6 months of GFD were associated with a significant increase of VD serum levels (from baseline 14.8 ± 5.39 ng/mL to 18.22 ± 5.67), but in several of them, VD levels remained in the abnormal range [81]. This seems to confirm the importance of adequate VD level monitoring in patients with CD, both at onset and during GFD. However, the optimal amount of VD supplementation to reach normal VD levels in CD patients is not established. Administration of patients with VD deficiency (<12 ng/mL) who, together with the GFD, were given 60,000 IU of VD per week during the first 3 months of treatment showed a significant increase in VD serum levels (from 9.45 ± 0.45 ng/mL to 13.53 ± 1.52 ng/mL), but no case reached normal VD values [81]. However, regardless of VD levels at onset or during GFD, most of the experts recommended that VD serum level monitoring be systematically performed in all subjects with CD, regardless of age, and VD deficiency, when documented, be corrected using the suggested dosage. The need for monitoring and VD administration to deficient CD patients has been confirmed by several scientific societies and institutions of different Western countries, such as the American College of Gastroenterology [82], the British Society of Gastroenterology [77,83], the North American Society for Pediatric Gastroenterology, Hepatology and Nutrition [78,84], and a group of Italian pediatric scientific societies [85].

## 6. Vitamin D and Inflammatory Bowel Disease

### 6.1. Epidemiology and Developmental Factors

Inflammatory bowel diseases (IBDs), including Crohn’s disease (CrD) and ulcerative colitis (UC), are a group of immune-mediated chronic conditions that are presumed to occur in genetically susceptible subjects due to a dysregulated intestinal immune response to one or more environmental factors [86]. It has been calculated that the incidence of IBD is approximately 0.1–16 cases per 100,000 person-years for CrD and 0.5–24.5 cases per 100,000 person-years for UC. Overall, the prevalence for IBD is 396 cases per 100,000 persons annually, with the highest rates of IBDs in developed countries, the lowest in developing regions and a time-trend significantly increasing over time [87].

Development of IBDs presupposes the coexistence of three different factors: genetic predisposition, dysregulated immune response, and one or more external factors triggering the immune response [88]. Although recently great importance has been ascribed to gut dysbiosis [89], the triggering factor that activates the abnormal immune response has yet to be identified. On the contrary, characteristics of genetic predisposition have been more precisely identified. In IBD patients, genetic variants in a total of 201 loci associated with the regulation of the intestinal barrier permeability and several functions of both the innate and the adaptive immunity have been identified [90,91]. These variants can impair all these functions and lead to a series of abnormal immune system responses, such as those found in IBD patients. CrD can be considered a prototype of T-helper (Th) 1-mediated diseases with increased levels of tumor necrosis factor-alpha (TNF-a), interferon gamma (IFN)-γ), and IL-12 and IL-1 in both the intestinal tissue and peripheral blood [70,71,72,73]. UC is usually viewed as a Th2-type condition because of the increased intestinal expression of excessive Th2 response, with upregulated secretion of IL-5, IL-4, IL-10, and IL-13 [92]. In both diseases, overexpression of pro-inflammatory cytokines was found to be associated with the initiation and progression of IBDs [93].

### 6.2. Relationships between Vitamin D and Inflammatory Bowel Disease Development

As previously reported, VD preserves the integrity of intestinal mucosa, maintains normal intestinal mucosa permeability, and modifies T cell cytokine responses from an inflammatory Th1 and Th17 phenotype towards a Th2 and a regulatory T cell phenotype [7]. This suggests that VD deficit may significantly influence IBD development and course, although it is not definitively clarified whether deficiency is the cause of IBDs or malabsorption following IBD development is associated with VD deficiency, and this influences IBD course [94]. VD deficiency can be detected in about 45–50% of UC patients, whereas it ranges from 35–100% in the case of CrD, and several studies have shown a potential relationship between VD serum levels and disease activity in IBDs [95]. Ham et al. studied this problem in 711 CrD patients and 764 UC patients [90]. A clinical disease activity score (CDAI) within 1 month of 25(OH)D measurement was analyzed in CrD patients, while the partial Mayo scores obtained on the day of 25(OH)D evaluation were used for UC patients. The study showed that VD deficiency was significantly associated with higher CDAI, partial Mayo scores, and C reactive protein (CRP) levels, confirming the role of VD in disease activity [96]. Furthermore, when the deficiency was severe, it was associated with severe manifestations of both CrD and UC, and it was an independent risk factor for intestinal surgery during the follow-up periods in both CrD and UC. Similar findings were reported by López-Muñoz et al., who found that serum 25(OH)D was inversely correlated with fecal calprotectin (FC) for CrD and UC, although correlated with CRP for UC patients [97]. The alteration of the laboratory indices was found to be greater, the more severe the symptoms were so that, considering together all of them, the authors could predict clinical characteristics of the patients and IBD severity with 80% accuracy. Finally, deficiency of 25(OH)D was associated with increased hospitalization, flare-ups, use of steroids, and escalating treatment [97]. These findings suggested that VD supplementation in IBD patients could be effective in reducing serum levels of inflammatory markers and in mitigating clinical manifestations of both CrD and UC.

### 6.3. Impact of Vitamin D Supplementation on Inflammatory Bowel Disease Course

Unfortunately, results of studies did not totally confirm these hypotheses. Administration of VD to CrD and UC patients generally resulted in a significant reduction of markers of inflammation, but the impact on clinical signs and symptoms of both diseases was generally poor or totally absent. Good examples in this regard are the studies by Sharifi et al. [98] and Bendix et al. [99]. Sharifi et al. reported that administration of a single intramuscular dose of 300 UI to mild-to-moderate UC patients was associated with a significant decrease of the inflammatory cytokines IL-12, IFN-γ, and TNF-a, without any reduction in IL-4 and IL-10 serum levels [92]. This confirmed the inhibitory effect of VD on Th1 immune responses and, through the reduction of TNF-a serum concentrations, seemed to suggest that VD could increase the chance of response to anti-TNF-a therapy used in severe IBD cases. Bendix et al. studied the impact of VD given orally (200,000 IU initially and a daily dose of 20,000 IU for 7 weeks) alone or with infliximab (5 mg/kg) in a group of 40 active CrD patients [93]. They found that the high dose VD supplementation, alone and combined with infliximab, decreased the IL-17A, IFNγ, and IL-10 expression. Particularly, the reduction in mucosal mRNA expression of IL-17 was considered a demonstration of a potential beneficial role of VD in CD patients, as IL-17 is considered a pivotal proinflammatory cytokine in the determination of inflammatory lesions of CrD [100].

However, the VD reduction in cytokine expression was not reflected in reduced disease activity scores, biopsy finding, and CRP or calprotectin values. Combined infliximab and VD treatment was not significantly superior to monotherapy with infliximab. On the other hand, the poor effect of VD supplementation in IBD patients is confirmed by some systematic reviews and meta-analysis of published studies. Li et al. analyzed 18 randomized clinical trials (RCTs) enrolling 908 patients and reported that VD could be effective in slightly reducing the disease relapse rate, whereas the laboratory tests were poorly influenced [101]. More detailed results were reported by Guo et al., who analyzed a total of 17 RCTs involving a total of 1127 patients with IBDs and showed that oral VD administration was safe, could be associated with a relevant reduction of CRP (standard mean difference [SMD] −0.33; 95% CI −0.61–−0.05), but had no effect on erythrocyte sedimentation rate levels (SMD 0.35; 95% CI −4.33–5.03), disease activity index (SMD −0.13; 95% CI −0.66–0.39), and relapse rate (RR 0.59; 95% CI 0.19–1.86) [102]. However, results of these meta-analyses cannot be considered definitive due to the differences among included studies in characteristics of the patients, the level of VD before supplementation, the amount and duration of VD supplementation, and the methods used to evaluate VD impact. Despite this, some experts suggest VD supplementation in IBD patients. Some information regarding the best dose of VD in IBD patients can be derived from the study by Goulart et al. [103]. Pooling the data collected with nine RCTs, they calculated that improvement of clinical score and patient quality of life were associated with 25(OH)D serum concentrations >20 ng/mL, and relapse could be avoided with 25(OH)D levels above 40 ng/mL. Oral daily doses from 1000 IU can raise VD levels above 20 ng/mL in UC or CrD patients with active disease, while doses close to 50,000 IU per week were sufficient to raise VD levels above 40 ng/mL. Even in this case, however, conclusions cannot be considered definitive due to the methodological limitations of many included studies, the use of very different VD supplementation (1000–300,000 IU), form of administration (oral liquid or capsules, or injectable) of VD and the duration of supplementation (1–12 months) [94] This could explain why scientific societies recommendations differ significantly regarding VD monitoring and supplementation in IBD patients. In the UK, there is no national recommendation, although oral VD supplementation is recommended when patients are treated with steroids. The European Crohn’s and Colitis Organization (ECCO) suggests VD to assure normal serum levels but does not stipulate what the normal range is. The American Gastroenterological Association (AGA) suggests vitamin D and calcium supplementation, particularly in patients at high risk of osteoporosis. Finally, the British Society of Gastroenterology, ECCO, and AGA guidelines regarding osteoporosis in IBD do not advocate routine measurement of serum vitamin D levels [103].

## 7. Conclusions

A potential impact of VD on CD and IBDs can be reasonably assumed based on the well-documented in vitro and in vivo VD activities on the gastrointestinal tract and the immune system. The evidence that VD can preserve intestinal mucosa from chemical and immunological damage and that VD modulation of the immune system functions can contrast the mechanisms that lead to the intestinal modifications characteristics of gastrointestinal autoimmune diseases has suggested that VD could play a role in controlling both development and course of CD and IBDs.

Unfortunately, in humans with these diseases, no definitive demonstration of these hypotheses has been reached. In many but not all the cases at diagnosis, low levels of VD have been demonstrated. Moreover, administration of VD in already diagnosed CD and IBD cases has not always significantly modified disease course. To make the evaluation of the clinical relevance of VD in autoimmune gastrointestinal diseases more difficult, several unsolved problems exist. The reference currently used to define VD serum concentrations remains debatable, mainly for the limitations of several methods used for 25(OH)D detection. Moreover, criteria used to define normal values of serum VD levels differ among experts, and this, together with several methodological limitations of the studies now performed to define the VD role, make collected data debatable. However, despite these relevant problems, most of the experts recommend monitoring of VD levels in patients with CD and IBDs and administration of supplements in patients with hypovitaminosis.

## Figures and Tables

**Table 1:** Vitamin D mechanisms in preserving the integrity of intestinal epithelial barrier.

Mechanism [36,37,38,39].
Regulation of colonic mucus
Preservation of epithelial integrity
Influence on gut microbiota composition and functions
Concentration-dependent mechanism of upregulation of tight junction protein expression
Suppression of release of zonulin

**Table 2:** Immunomodulating activity of vitamin D on immune systems.

Innate Immunity [42,43,44,45,46]
Stimulation of the production by neutrophils, macrophages, and cells liningepithelial surfaces of CAMP and DEFB4
Increase in the antimicrobial effect against pathogens
Induction of the intracellular pathogen recognition receptor NOD2
Enhancement in transcription of CAMP and DEFB4
Suppression of hepcidin antimicrobial peptide expression
Reduction in ferroportin-mediated export of intracellular iron
**Adaptive Immunity** [47]
Differentiation and maturation of DC
Expression on monocytes of molecules involved in antigen capture
Reduction in pro-inflammatory Th1 response
Increase anti-inflammatory Th2 response
Increase in T regulatory cells
Limitation of the number of CD4+ T cells

CAMP, cathelicidin; DEFB4, β-defensin 2; DC, dendritic cells (DC).

## Data Availability

All the data are included in the manuscript.

## References

[1] Laird E., Ward M., McSorley E., Strain J.J., Wallace J. (2010). Vitamin D and bone health: Potential mechanisms. Nutrients.

[2] Bouillon R., Marcocci C., Carmeliet G., Bikl D., White J.H., Dawson-Hughes B. (2019). Skeletal and Extraskeletal Actions of Vitamin D: Current Evidence and Outstanding Questions. Endocr. Rev..

[3] Charoenngam N., Holick M.F. (2020). Immunologic Effects of Vitamin D on Human Health and Disease. Nutrients.

[4] Bishop E., Ismailova A., Dimeloe S., Hewison M., White J.H. (2020). Vitamin D and Immune Regulation: Antibacterial, Antiviral, Anti-Inflammatory. JBMR Plus.

[5] Mailhot G., White J.H. (2020). Vitamin D and Immunity in Infants and Children. Nutrients.

[6] Akimbekov N.S., Digel I., Sherelkhan D.K., Lutfor A.B., Razzaque M.S. (2020). Vitamin D and the Host-Gut Microbiome: A Brief Overview. Acta Histochem. Cytochem..

[7] Jolliffe D.A., Camargo C.A., Sluyter J.D., Aglipay M., Aloia J.F., Ganmaa D., Bergman P., Bischoff-Ferrari H.A., Borzutzky A., Damsgaard C.T. (2021). Vitamin D supplementation to prevent acute respiratory infections: A systematic review and meta-analysis of aggregate data from randomised controlled trials. Lancet Diabetes Endocrinol..

[8] Jäpelt R.B., Jakobsen J. (2013). Vitamin D in plants: A review of occurrence, analysis, and biosynthesis. Front. Plant Sci..

[9] Saponaro F., Saba A., Zucchi R. (2020). An Update on Vitamin D Metabolism. Int. J. Mol. Sci..

[10] McKenzie R., Scragg R., Liley B., Johnston P., Wishart J., Stewart A., Prematunga R. (2012). Serum 25-hydroxyvitamin-D responses to multiple UV exposures from solaria: Inferences for exposure to sunlight. Photochem. Photobiol. Sci..

[11] Rusińska A., Płudowski P., Walczak M., Borszewska-Kornacka M.K., Bossowski A., Chlebna-Sokół D., Czech-Kowalska J., Dobrzańska A., Franek E., Helwich E. (2018). Vitamin D Supplementation Guidelines for General Population and Groups at Risk of Vitamin D Deficiency in Poland-Recommendations of the Polish Society of Pediatric Endocrinology and Diabetes and the Expert Panel With Participation of National Specialist Consultants and Representatives of Scientific Societies-2018 Update. Front Endocrinol.

[12] Zhu J.G., Ochalek J.T., Kaufmann M., Jones G., Deluca H.F. (2013). CYP2R1 is a major, but not exclusive, contributor to 25-hydroxyvitamin D production in vivo. Proc. Natl. Acad. Sci. USA.

[13] Christakos S., Dhawan P., Verstuyf A., Verlinden L., Carmeliet G. (2016). Vitamin D: Metabolism, molecular mechanism of action, and pleiotropic effects. Physiol. Rev..

[14] Kongsbak M., Levring T.B., Geisler C., von Essen M.R. (2013). The vitamin d receptor and T cell function. Front Immunol..

[15] Wang Y., Zhu J., DeLuca H.F. (2012). Where is the vitamin D receptor?. Arch Biochem. Biophys..

[16] Zenata O., Vrzal R. (2017). Fine tuning of vitamin D receptor (VDR) activity by post-transcriptional and post-translational modifications. Oncotarget.

[17] Sempos C.T., Heijboer A.C., Bikle D.D., Bollerslev J., Bouillon R., Brannon P.M., DeLuca H.F., Jones G., Munns C.F., Bilezikian J.P. (2018). Vitamin D assays and the definition of hypovitaminosis D. Results from the First International onference on Controversies in Vitamin D. Br. J. Clin. Pharmacol..

[18] LeFevre M.L. (2015). Screening for vitamin deficiency in adults: U.S. Preventive Services Task Force recommendation statement. Ann. Intern. Med..

[19] Bikle D.D., Schwartz J. (2019). Vitamin D Binding Protein, Total and Free Vitamin D Levels in Different Physiological and Pathophysiological Conditions. Front. Endocrinol..

[20] Cashman K.D., Hayes A., Galvin K., Merkel J., Jones G., Kaufmann M., Hoofnagle A.N., Carter G.D., Durazo-Arvizu R.A., Sempos C.T. (2015). Significance of serum 24,25-dihydroxyvitamin D in the assessment of vitamin D status: A double-edged sword?. Clin. Chem..

[21] Binkley N., Dawson-Hughes B., Durazo-Arvizu R., Thamm M., Tian L., Merkel J.M., Jones J.C., Carter G.D., Sempos C.T. (2017). Vitamin D measurement standardization: The way out of the chaos. J. Steroid. Biochem. Mol. Biol..

[22] Tai S.S., Bedner M., Phinney K.W. (2010). Development of a candidate reference measurement procedure for the determination of 25-hydroxyvitamin D3 and 25-hydroxyvitamin D2 in human serum using isotope-dilution liquid chromatography-tandem mass spectrometry. Anal. Chem..

[23] Institute of Medicine, Food and Nutrition Board (2010). Dietary Reference Intakes for Calcium and Vitamin D.

[24] Holick M.F., Binkley N.C., Bischoff-Ferrari H.A., Gordon C.M., Hanley D.A., Heaney R.P., Murad M.H., Weaver C.M., Endocrine Society (2011). Evaluation, treatment, and prevention of vitamin D deficiency: An Endocrine Society Clinical Practice Guideline. J. Clin. Endocrinol. Metab..

[25] EFSA Panel on Dietetic Products, Nutrition and Allergies (NDA) (2012). Scientific Opinion on the Tolerable Upper Intake Level of vitamin D. EFSA J..

[26] Chung M., Balk E.M., Brendel M., Ip S., Lau J., Lee J., Lichtenstein A., Patel K., Raman G., Tatsioni A. (2009). Vitamin D and calcium: A systematic review of health outcomes. Evid. Rep. Technol. Assess (Full Rep)..

[27] Principi N., Bianchini S., Baggi E., Esposito S. (2013). Implications of maternal vitamin D deficiency for the fetus, the neonate and the young infant. Eur. J. Nutr..

[28] Beauchesne A.R., Cara K.C., Krobath D.M., Penkert L.P., Shertukde S.P., Cahoon D.S., Prado B., Li R., Yao Q., Huang J. (2022). Vitamin D intakes and health outcomes in infants and preschool children: Summary of an evidence report. Ann. Med..

[29] Fasano A., Shea-Donohue T. (2005). Mechanisms of disease: The role of intestinal barrier function in the pathogenesis of gastrointestinal autoimmune diseases. Nat. Clin. Pract. Gastroenterol. Hepatol..

[30] Zhu W., Yan J., Zhi C., Zhou Q., Yuan X. (2019). 1,25(OH)2D3 deficiency-induced gut microbial dysbiosis degrades the colonic mucus barrier in Cyp27b1 knockout mouse model. Gut Pathog..

[31] Kühne H., Hause G., Grundmann S.M., Schutkowski A., Brandsch C., Stangl G.I. (2016). Vitamin D receptor knockout mice exhibit elongated intestinal microvilli and increased ezrin expression. Nutr. Res..

[32] Schäffler H., Herlemann D.P., Klinitzke P., Berlin P., Kreikemeyer B., Jaster R., Lamprecht G. (2018). Vitamin D administration leads to a shift of the intestinal bacterial composition in Crohn’s disease patients, but not in healthy controls. J. Dig. Dis..

[33] Fasano A. (2012). Zonulin, regulation of tight junctions, and autoimmune diseases. Ann. N. Y. Acad. Sci..

[34] Chibbar R., Dieleman L.A. (2019). The Gut Microbiota in Celiac Disease and probiotics. Nutrients.

[35] Mentella M.C., Scaldaferri F., Pizzoferrato M., Gasbarrini A., Miggiano G.A.D. (2019). The Association of Disease Activity, BMI and Phase Angle with Vitamin D Deficiency in Patients with IBD. Nutrients.

[36] Zhao H., Zhang H., Wu H., Li H., Liu L., Guo J., Li C., Shih D.Q., Zhang X. (2012). Protective role of 1, 25 (OH) 2 vitamin D 3 in the mucosal injury and epithelial barrier disruption in DSS-induced acute colitis in mice. BMC Gastroenterol..

[37] Chen S.-W., Ma Y.-Y., Zhu J., Zuo S., Zhang J.-L., Chen Z.-Y., Chen G.-W., Wang X., Pan Y.-S., Liu Y.-C. (2015). Protective effect of 1, 25-dihydroxyvitamin D3 on ethanol-induced intestinal barrier injury both in vitro and in vivo. Toxicol. Lett..

[38] Chen S., Zhu J., Chen G., Zuo S., Zhang J., Chen Z., Wang X., Li J., Liu Y., Wang P. (2015). 1,25-Dihydroxyvitamin D3 preserves intestinal epithelial barrier function from TNF-α induced injury via suppression of NF-kB p65 mediated MLCK-P-MLC signaling pathway. Biochem. Biophys. Res. Commun..

[39] Zhang Y.-G., Wu S., Lu R., Zhou D., Zhou J., Carmeliet G., Petrof E., Claud E.C., Sun J. (2015). Tight junction CLDN2 gene is a direct target of the vitamin D receptor. Sci. Rep..

[40] Chatterjee I., Zhang Y., Zhang J., Lu R., Xia Y., Sun J. (2021). Overexpression of Vitamin D Receptor in Intestinal Epithelia Protects Against Colitis via Upregulating Tight Junction Protein Claudin 15. J. Crohns. Colitis..

[41] Zhang Y.-G., Lu R., Xia Y., Zhou D., Petrof E., Claud E.C., Sun J. (2019). Lack of Vitamin D Receptor Leads to Hyperfunction of Claudin-2 in Intestinal Inflammatory Responses. Inflamm. Bowel Dis..

[42] Weber G., Heilborn J.D., Jimenez C.I.C., Hammarsjö A., Törmä H., Ståhle M. (2005). Vitamin D induces the antimicrobial protein hCAP18 in human skin. J. Investig. Dermatol..

[43] Bals R., Wang X., Zasloff M., Wilson J.M. (1998). The peptide antibiotic LL37/hCAP-18 is expressed in epithelia of the human lung where it has broad antimicrobial activity at the airway surface. Proc. Natl. Acad. Sci. USA.

[44] Gallo R.L., Kim K.J., Bernfield M., Kozak C.A., Zanetti M., Merluzzi L., Gennaro R. (1997). Identification of CRAMP, a cathelin-related antimicrobial peptide expressed in the embryonic and adult mouse. J. Biol. Chem..

[45] Chun R.F., Liu P.T., Modlin R.L., Adams J.S., Hewison M. (2014). Impact of vitamin D on immune function: Lessons learned from genome-wide analysis. Front Physiol..

[46] Bacchetta J., Zaritsky J.J., Sea J.L., Chun R., Lisse T.S., Zavala K., Nayak A., Wesseling-Perry K., Westerman M., Hollis B.W. (2014). Suppression of iron-regulatory hepcidin by vitamin D. J. Am. Soc. Nephrol..

[47] Bikle D.D. (2022). Vitamin D Regulation of Immune Function. Curr. Osteoporos. Rep..

[48] Lebwohl B., Sanders D.S., Green P.H.R. (2018). Coeliac disease. Lancet.

[49] Klöck C., Diraimondo T.R., Khosla C. (2012). Role of transglutaminase 2 in celiac disease pathogenesis. Semin. Immunopathol..

[50] Corazza G.R., Villanacci V., Zambelli C., Milione M., Luinetti O., Vindigni C., Chioda C., Albarello L., Bartolini D., Donato F. (2007). Comparison of the interobserver reproducibility with different histologic criteria used in celiac disease. Clin. Gastroenterol. Hepatol..

[51] Andersen D.H. (1947). Celiac syndrome: The relationship of celiac disease, starch intolerance, and steatorrhea. J. Pediatr..

[52] Nardecchia S., Auricchio R., Discepolo V., Troncone R. (2019). Extra-Intestinal Manifestations of Coeliac Disease in Children: Clinical Features and Mechanisms. Front. Pediatr..

[53] Ludvigsson J.F., Leffler D.A., Bai J.C., Biagi F., Fasano A., Green P.H.R., Hadjivassiliou M., Kaukinen K., Kelly C.P., Leonard J.N. (2013). The Oslo definitions for coeliac disease and related terms. Gut.

[54] Lauret E., Rodrigo L. (2013). Celiac disease and autoimmune-associated conditions. Biomed. Res. Int..

[55] Pecora F., Persico F., Gismondi P., Fornaroli F., Iuliano S., de’Angelis G.L., Esposito S. (2020). Gut Microbiota in Celiac Disease: Is There Any Role for Probiotics?. Front. Immunol..

[56] Gundemir S., Colak G., Tucholski J., Johnson G.V. (2012). Transglutaminase 2: A molecular Swiss army knife. Biochim. Biophys. Acta..

[57] Singh P., Arora A., Strand T.A., Leffler D.A., Catassi C., Green P.H., Kelly C.P., Ahuja V., Makharia G.K. (2018). Global Prevalence of Celiac Disease: Systematic Review and Meta-analysis. Clin. Gastroenterol. Hepatol..

[58] Virta L.J., Kaukinen K., Collin P. (2009). Incidence and prevalence of diagnosed coeliac disease in Finland: Results of effective case finding in adults. Scand. J. Gastroenterol..

[59] Liu E., Dong F., Barón A.E., Taki I., Norris J.M., Frohnert B.I., Hoffenberg E.J., Rewers M. (2017). High incidence of celiac disease in a long-term study of adolescents with susceptibility genotypes. Gastroenterology.

[60] King J.A., Jeong J., Underwood F.E., Quan J., Panaccione N., Windsor J.W., Coward S., Debruyn J., Ronksley P.E., Shaheen A.-A. (2020). Incidence of Celiac Disease Is Increasing Over Time: A Systematic Review and Meta-analysis. Am. J. Gastroenterol..

[61] Li M., Yu L., Tiberti C., Bonamico M., Taki I., Miao D., Murray J.A., Rewers M.J., Hoffenberg E.J., Agardh D. (2009). A report on the International Transglutaminase Autoantibody Workshop for Celiac Disease. Am. J. Gastroenterol..

[62] Green P.H., Lebwohl B., Greywoode R. (2015). Celiac disease. J. Allergy Clin. Immunol..

[63] Verdu E.F., Schuppan D. (2021). Co-factors, Microbes, and Immunogenetics in Celiac Disease to Guide Novel Approaches for Diagnosis and Treatment. Gastroenterology.

[64] Pecora F., Persico F., Argentiero A., Neglia C., Esposito S. (2020). The Role of Micronutrients in Support of the Immune Response against Viral Infections. Nutrients.

[65] Unalp-Arida A., Ruhl C.E., Choung R.S., Brantner T.L., Murray J.A. (2017). Lower Prevalence of Celiac Disease and Gluten-Related Disorders in Persons Living in Southern vs Northern Latitudes of the United States. Gastroenterol.

[66] Ivarsson A., Hernell O., Nyström L., Persson L.A. (2003). Children born in the summer have increased risk for coeliac disease. J. Epidemiol. Community Health.

[67] Krupa-Kozak U. (2014). Pathologic bone alterations in celiac disease: Etiology, epidemiology, and treatment. Nutrition.

[68] Delbue D., Cardoso-Silva D., Branchi F., Itzlinger A., Letizia M., Siegmund B., Schumann M. (2019). Celiac Disease Monocytes Induce a Barrier Defect in Intestinal Epithelial Cells. Int. J. Mol. Sci..

[69] Sander G.R., Cummins A.G., Henshall T., Powell B.C. (2005). Rapid disruption of intestinal barrier function by gliadin involves altered expression of apical junctional proteins. FEBS Lett..

[70] Dong S., Singh T.P., Wei X., Yao H., Wang H. (2018). Protective Effect of 1,25-Dihydroxy Vitamin D3 on Pepsin-Trypsin-Resistant Gliadin-Induced Tight Junction Injuries. Dig. Dis. Sci..

[71] Lionetti E., Catassi C. (2011). New clues in celiac disease epidemiology, pathogenesis, clinical manifestations, and treatment. Int. Rev. Immunol..

[72] Dewar D., Pereira S.P., Ciclitira P.J. (2004). The pathogenesis of coeliac disease. Int. J. Biochem. Cell Biol..

[73] Ferretti G., Bacchetti T., Masciangelo S., Saturni L. (2012). Celiac disease, inflammation and oxidative damage: A nutrigenetic approach. Nutrients.

[74] Villanueva J., Maranda L., Nwosu B.U. (2012). Is vitamin D deficiency a feature of pediatric celiac disease?. J. Pediatr. Endocrinol. Metab..

[75] Lerner A., Shapira Y., Agmon-Levin N., Pacht A., Shor D.B.-A., López H.M., Sanchez-Castanon M., Shoenfeld Y. (2012). The clinical significance of 25OH-Vitamin D status in celiac disease. Clin. Rev. Allergy Immunol..

[76] Lu C., Zhou W., He X., Zhou X., Yu C. (2021). Vitamin D status and vitamin D receptor genotypes in celiac disease: A meta-analysis. Crit. Rev. Food Sci. Nutr..

[77] Lionetti E., Galeazzi T., Dominijanni V., Acquaviva I., Catassi G.N., Iasevoli M., Malamisura B., Catassi C. (2021). Lower Level of Plasma 25-Hydroxyvitamin D in Children at Diagnosis of Celiac Disease Compared with Healthy Subjects: A Case-Control Study. J. Pediatr..

[78] Akhshayaa G., Seth A., Kumar P., Jain A. (2021). Prevalence and management of vitamin D deficiency in children with newly diagnosed coeliac disease: Cohort study. Paediatr. Int. Child Health.

[79] Wahab P.J., Meijer J.W., Mulder C.J. (2002). Histologic follow-up of people with celiac disease on a gluten-free diet: Slow and incomplete recovery. Am. J. Clin. Pathol..

[80] Tursi A., Brandimarte G., Giorgetti G.M., Elisei W., Inchingolo C.D., Monardo E., Aiello F. (2006). Endoscopic and histological findings in the duodenum of adults with celiac disease before and after changing to a gluten-free diet: A 2-year prospective study. Endoscopy.

[81] Verma A., Lata K., Khanna A., Singh R., Sachdeva A., Jindal P., Yadav S. (2022). Study of effect of gluten-free diet on vitamin D levels and bone mineral density in celiac disease patients. J. Family Med. Prim. Care.

[82] Rubio-Tapias A., Hill I.D., Kelly C.P., Calderwood A.H., Murray J.A. (2013). American College of Gastroenterology clinical guideline: Diagnosis and management of celiac disease. Am. J. Gastroenterol..

[83] Ludvigsson J.F., Bai J.C., Biagi F., Card T.R., Ciacci C., Ciclitira P.J., Green P.H.R., Hadjivassiliou M., Holdoway A., van Heel D.A. (2014). Diagnosis and management of adult coeliac disease: Guidelines from the British Society of Gastroenterology. Gut.

[84] Hill I.D., Fasano A., Guandalini S., Hoffenberg E., Levy J., Reilly N., Verma R. (2016). NASPGHAN clinical report on the diagnosis and treatment of gluten-related disorders. J. Pediatr. Gastroenterol. Nutr..

[85] Saggese G., Vierucci F., Prodam F., Cardinale F., Cetin I., Chiappini E., De’ Angelis G.L., Massari M., Miraglia Del Giudice E., Miraglia Del Giudice M. (2018). Vitamin D in pediatric age: Consensus of the Italian Pediatric Society and the Italian Society of Preventive and Social Pediatrics, jointly with the Italian Federation of Pediatricians. Ital. J. Pediatr..

[86] Park S.J., Kim W.H., Cheon J.H. (2014). Clinical characteristics and treatment of inflammatory bowel disease: A comparison of Eastern and Western perspectives. World J. Gastroenterol..

[87] Centers for Disease Control and Prevention Inflammatory Bowel Disease (IBD). http://www.cdc.gov/ibd/#epidIBD.

[88] Boros É., Hegedűs Z., Kellermayer Z., Balogh P., Nagy I. (2022). Global alteration of colonic microRNAome landscape associated with inflammatory bowel disease. Front. Immunol..

[89] Brusaferro A., Cavalli E., Farinelli E., Cozzali R., Principi N., Esposito S. (2019). Gut dysbiosis and paediatric Crohn’s disease. J. Infect..

[90] Mehta M., Ahmed S., Dryden G. (2017). Immunopathophysiology of inflammatory bowel disease: How genetics link barrier dysfunction and innate immunity to inflammation. Innate Immun..

[91] Muzes G., Molnár B., Tulassay Z., Sipos F. (2012). Changes of the cytokine profile in inflammatory bowel diseases. World J. Gastroenterol..

[92] de Mattos B.R., Garcia M.P., Nogueira J.B., Paiatto L.N., Albuquerque C.G., Souza C.L., Fernandes L.G., Tamashiro W.M., Simioni P.U. (2015). Inflammatory Bowel Disease: An Overview of Immune Mechanisms and Biological Treatments. Mediators Inflamm..

[93] Lee S.H., Kwon J.E., Cho M.L. (2018). Immunological pathogenesis of inflammatory bowel disease. Intest. Res..

[94] Goulart R.A., Barbalho S.M. (2022). Can vitamin D induce remission in patients with inflammatory bowel disease?. Ann. Gastroenterol..

[95] Wu Z., Liu D., Deng F. (2022). The Role of Vitamin D in Immune System and Inflammatory Bowel Disease. J. Inflam. Res..

[96] Ham N.S., Hwang S.W., Oh E.H., Kim J., Lee H.S., Park S.H., Yang D.H., Ye B.D., Byeon J.S., Myung S.J. (2021). Influence of Severe Vitamin D Deficiency on the Clinical Course of Inflammatory Bowel Disease. Dig. Dis. Sci..

[97] López-Muñoz P., Beltrán B., Sáez-González E., Alba A., Nos P., Iborra M. (2019). Influence of Vitamin D Deficiency on Inflammatory Markers and Clinical Disease Activity in IBD Patients. Nutrients.

[98] Sharifi A., Vahedi H., Nedjat S., Rafiei H., Hosseinzadeh-Attar M.J. (2019). Effect of single-dose injection of vitamin D on immune cytokines in ulcerative colitis patients: A randomized placebo-controlled trial. APMIS.

[99] Bendix M., Dige A., Jørgensen S.P., Dahlerup J.F., Bibby B.M., Deleuran B., Agnholt J. (2020). Decrease in Mucosal IL17A, IFNγ and IL10 Expressions in Active Crohn’s Disease Patients Treated with High-Dose Vitamin Alone or Combined with Infliximab. Nutrients.

[100] Jiang W., Su J., Zhang X., Cheng X., Zhou J., Shi R., Zhang H. (2014). Elevated levels of Th17 cells and Th17-related cytokines are associated with disease activity in patients with inflammatory bowel disease. Inflamm. Res..

[101] Li J., Chen N., Wang D., Zhang J., Gong X. (2018). Efficacy of vitamin D in treatment of inflammatory bowel disease: A meta-analysis. Medicine.

[102] Guo Y., Zhang T., Wang Y., Liu R., Chang M., Wang X. (2021). Effects of oral vitamin D supplementation on inflammatory bowel disease: A systematic review and meta-analysis. Food Funct..

[103] Fletcher J., Swift A., Hewison M., Cooper S.C. (2020). Screening and Treatment of Vitamin D Deficiency in UK Patients with Crohn’s Disease: Self-Reported Practice among Gastroenterologists. Nutrients.

